# A Suspected Case of COVID-19-Induced Immunosuppression

**DOI:** 10.7759/cureus.32227

**Published:** 2022-12-05

**Authors:** Kofi Seffah, Walter Y Agyeman

**Affiliations:** 1 Internal Medicine, Piedmont Athens Regional Medical Center, Athens, USA

**Keywords:** immune, adaptive, cytokine, vaccination, covid, immunocompromised, cryptococcal meningitis

## Abstract

While COVID-19 has gained popularity as a pandemic and as a cause of pulmonary-systemic disease, the condition continues to evolve our knowledge and understanding of immunology and medicine through its myriad clinical presentations. This article features a previously healthy 65-year-old female who presented with sudden features of cryptococcal meningitis, the progression of which raises questions as to what role the virus plays in the innate, adaptive, and overall host factors leading to immunosuppression.

## Introduction

COVID-19 continues to contribute significantly to healthcare outcomes across the world. The nature of the disease, its pathogenesis, and the extent of its effects are still being studied.

We have a 65-year-old female who presented positive for COVID-19 and was diagnosed with cryptococcal meningitis. While her diagnosis in itself was unusual in an immunocompetent patient, her disease progression was even more baffling, as would be found in an immunocompromised patient. We would like to present this as proof for medical consideration of COVID-19 as a significant cause of immunosuppression and a risk factor for opportunistic infections [1].

## Case presentation

A 65-year-old female with a past medical history of hypertension and schizophrenia was admitted to the hospital on February 11, 2022, following a diagnosis of altered mental status secondary to cryptococcal meningitis. She presented with no respiratory symptoms.

Her initial evaluation for an underlying cause of immunosuppression was remarkably unrevealing. The patient had no history of recurrent illness or admissions. She did not have a diagnosis of chronic obstructive pulmonary disease (COPD), pulmonary fibrosis, or lung infections. She had not previously been diagnosed with diabetes. She did not have chronic liver disease, chronic kidney disease, or heart disease. She had not been exposed to steroids, not even for the treatment of COVID-19. She was tested and shown to be HIV-negative. She was not a transplant/organ recipient. She had not received any Food and Drug Administration (FDA)-approved antiviral or immunomodulatory treatment for COVID-19. She was not on any immunosuppressive medications.

She, however, tested positive for COVID-19 on February 3, 2022, from the referral site, and although her presenting symptoms were not supportive of the respiratory distress that characterizes this viral infection, her clinical course declined steadily following the said diagnosis. Based on her deteriorating mental state, cerebrospinal fluid (CSF) was obtained, which showed positive cryptococcal antigens. Her white blood cell count was 18.5/mcL with 90% neutrophils. She was immediately started on antifungals. It is important to note that the patient had received the COVID-19 vaccine, with three documented shots (March, April, and November 2021). In addition, records indicate that she was up to date with all vaccination, including influenza, pneumococcal, and tetanus, diphtheria, and pertussis (Tdap) vaccines.

Her presentation was more concerning for cryptococcal meningitis, despite the COVID-19 positivity. She did not have respiratory symptoms. Treatment was directed to this on admission with amphotericin B and flucytosine. Despite appropriate treatment, her clinical course deteriorated dramatically, akin to the progression of the disease in the immunosuppressed. She progressed to require repeated lumbar punctures, with worsening neurological function despite optimal treatment. The management team included infectious disease, cardiology, intensivist, neurology, and neurosurgical specialties. No treatment was offered for COVID-19, as this at the time was not deemed consequential in her overall clinical picture. The COVID-19 test was repeated on March 31, 2022, and was found to be negative. However, repeated tests to elucidate the risk factors that predisposed this patient to a condition mainly associated with the immunocompromised proved elusive.

Throughout her hospital stay, the patient continued to decline, becoming barely responsive and requiring repeated lumbar punctures for her elevated intracranial pressure and declining mental status. No clinical recovery of her mental state was noted throughout her stay, and comfort care was opted for after it was agreed with the family that more interventions were unlikely to yield any improvement. She spent a total of 48 days on admission.

## Discussion

The first documented case of COVID-19 was recorded in the Wuhan province in China in December 2019, with the first case reported in the United States in January of the following year [1,2]. SARS-CoV-2, the virus responsible for COVID-19, is a single, positive-strand RNA virus. Spike proteins on the surface of the virus have been identified as key antigenic components in host invasion, targeting angiotensin-converting enzyme-2 (ACE-2) receptors in the host, using receptor-mediated endocytosis as the main process of viral entry to the host cells [3,4]. The proposed mechanism by which the virus invades the host’s immune system involves the evasion of the innate immune system [5].

A significant number of people who are infected with the COVID-19 virus do not show symptoms. Most infected patients only show mild upper respiratory symptoms. Some individuals, however, show severe symptoms, the severity of which is noted to be high among the immunosuppressed [6].

For the purposes of our article, we will employ the description of the response of the immune system to the virus as adaptive and maladaptive. An increase in leukocytes in response to the invasion of the virus, with mild symptoms and eventual resolution, is seen as adaptive and akin to the clinical response to COVID-19 vaccination [5]. However, more evidence is emerging that a dysregulated cytokine release leading to a cytokine storm may be responsible for a maladaptive response to the virus in which there is damage to the tissue of the lungs and kidneys primarily from the host immune response rather than from direct viral injury [5,7-9]. To add to this, lymphocyte depletion has been documented as an additional finding in severe COVID-19 infection, implying immunosuppression [10]. We postulate that COVID-19 places patients at high risk of opportunistic infections.

Our patient was diagnosed with COVID-19 about the same time she presented with her symptoms that would eventually be diagnosed as cryptococcal meningitis. She was up to date with vaccination, including two shots of COVID-19 vaccines the previous year. It is, however, not uncommon for individuals vaccinated for COVID-19 to contract the virus.

Cryptococcal meningitis is an opportunistic infection [11]. It has, however, been documented to occur in the immunocompetent as well [12-14]. In clinical practice, it behooves the practitioner to identify what risk factors are at play that led to the patient’s infection. No such factors were identified in this patient. Her COVID-19 positivity was not considered to be a predisposing factor for her infection with *Cryptococcus*, and no adjunct therapy was offered as part of management since she exhibited no symptoms. Her vaccination status was reassuring, which may also have influenced the need to pay less attention to her COVID-19 positivity. People vaccinated for COVID-19 are known to have milder forms of infection [15]. This may, however, not be true for all cases. Our patient cannot be said to have had ongoing (symptoms more than 4-12 weeks following initial infection) or long COVID-19 recovery (symptoms persisting beyond 12 weeks of initial infection) either as she had never been documented to have contracted COVID-19 prior to her final admission. There was also no evidence of post-COVID-19 syndrome, where there is virological clearance (negative polymerase chain reaction (PCR) test) but with various symptoms persisting [16-18].

This article seeks to highlight that even in subclinical presentations (where COVID-19 testing is positive and there are mild or no symptoms), maladaptive immune responses may still be at play, predisposing infected patients to opportunistic infections, as in this patient, or potentially trigger underlying and previously indolent ailments [10]. While we still strongly recommend vaccination, social distancing, handwashing, and using facemasks, we would like to point out that even the seemingly unaffected may be at increased risk of other infectious diseases, whether or not they have previously documented underlying immune suppression or vaccination [19].

## Conclusions

Research into the biomolecular pathogenesis of COVID-19 is still ongoing, and while this article is insufficient to raise alarm on an already globally exhausting disease entity, with at least 0.8 million deaths within the first year, we seek to highlight an important research agenda on the link between immunosuppression and COVID-19, a globally deleterious disease.

We would also like to propose this COVID-19 syndrome of induced immunosuppression as a separate entity to be considered in the workup of future patients. This may lend support for developing more robust guidelines for managing the apparently less clinically virulent forms of the infection.
